# Impact of depression and antidepressant use on clinical outcomes of hepatitis B and C: a population-based study

**DOI:** 10.1097/HC9.0000000000000062

**Published:** 2023-02-14

**Authors:** Abdel Aziz Shaheen, Gilaad G. Kaplan, Keith A. Sharkey, B. Cord Lethebe, Mark G. Swain

**Affiliations:** 1 Division of Gastroenterology and Hepatology, Department of Medicine, Cumming School of Medicine, University of Calgary, Calgary, Alberta, Canada; 2 Snyder Institute for Chronic Diseases, Cumming School of Medicine, University of Calgary, Calgary, Alberta, Canada; 3 Department of Physiology and Pharmacology, University of Calgary, Calgary, Alberta, Canada; 4 Hotchkiss Brain Institute, Cumming School of Medicine, University of Calgary, Calgary, Alberta, Canada

## Abstract

Depression is common in patients with chronic viral hepatitis. We evaluated the impact of major depressive disorder (MDD) and antidepressant use on survival among patients with HBV and HCV.

**Methods:**

We used The Health Improvement Network database, the largest medical database in the UK, to identify incident HBV (n=1401) and HCV (n=1635) in patients between 1986 and 2017. Our primary composite outcome was the development of decompensated cirrhosis or death. MDD and each class of antidepressants were assessed in multivariate Cox proportional hazards models. Models were adjusted for age, sex, and clinical comorbidities.

**Results:**

The prevalence of MDD among HCV patients was higher compared with HBV patients (23.5% vs. 9.0%, *p*<0.001, respectively). Similarly, HCV patients were more likely to use antidepressants (59.6%) compared with HBV patients (27.1%), *p*>0.001. MDD was not an independent predictor for decompensated cirrhosis-free survival or mortality. However, the use of tricyclic and tetracyclic antidepressants (TCAs) was associated with poor decompensated cirrhosis-free survival in HBV and HCV cohorts (adjusted HR: 1.80, 95% CI, 1.00–3.26 and 1.56, 95% CI, 1.13–2.14, respectively). Both TCAs in the HBV cohort and selective serotonin reuptake inhibitors among the HCV cohort were associated with poor overall survival (adjusted HR: 2.18, 95% CI, 1.16–4.10; 1.48, 95% CI, 1.02–2.16, respectively).

**Conclusions:**

Although prevalent among viral hepatitis patients, MDD did not affect disease progression or survival in either HBV or HCV cohorts. TCA use was associated with poor decompensated cirrhosis-free survival. Therefore, its use should be further studied among viral hepatitis patients.

## INTRODUCTION

The lifetime risk for developing major depressive disorder (MDD) is estimated to be between 15% and 18%,^1^ and MDD is a leading cause of morbidity, disability, and poor clinical outcomes worldwide.^2^ MDD is a risk factor for disease progression and poor survival in various diseases, including cancer, diabetes mellitus, cardiovascular disease, and immune-mediated diseases.^3^–^5^

HBV and HCV represent the most common causes of chronic hepatitis worldwide.^6^ Depressive symptoms are highly prevalent among patients with HCV and HBV but seem to be more common in those with HCV.^7^,^8^ A recent large meta-analysis reported an overall MDD rate of 24.5% in HCV-infected individuals,^9^ with up to two thirds of depressed HCV-infected patients taking antidepressants.^10^ In contrast, the rates of depressive symptoms and MDD in HBV-infected patients have been reported to be 12.4% and 6.4%, respectively.^11^,^12^ Interestingly, depressive symptoms have been linked to increased liver-related mortality in HBV-infected people.^11^

Antidepressant usage was associated with improved survival among patients with myocardial infarction and primary biliary cholangitis.^13^,^14^

There is limited knowledge on the longitudinal impact of MDD or antidepressants and clinical outcomes among patients with HBV or HCV in the general population. Therefore, we addressed this gap by studying the impact of MDD and antidepressant use on disease progression and survival among patients with incident HBV or HCV diagnosis using a large representative population-based database.

## METHODS

### Study design and patient data source

We used the UK-based electronic medical records database, The Health Improvement Network (THIN), to conduct a retrospective cohort study.^15^ THIN consists of prospectively gathered data from >14 million individuals, representative of the general UK population. Patients enrolled in THIN have demographic and mortality distributions that are similar to the general UK population.^16^,^17^ The THIN database identifies demographic and disease or clinical data as “Read codes.”^15^ Validation of the THIN database Read codes has been reported in chronic liver diseases, including viral hepatitis.^18^–^20^

### Study population

**FIGURE 1:**
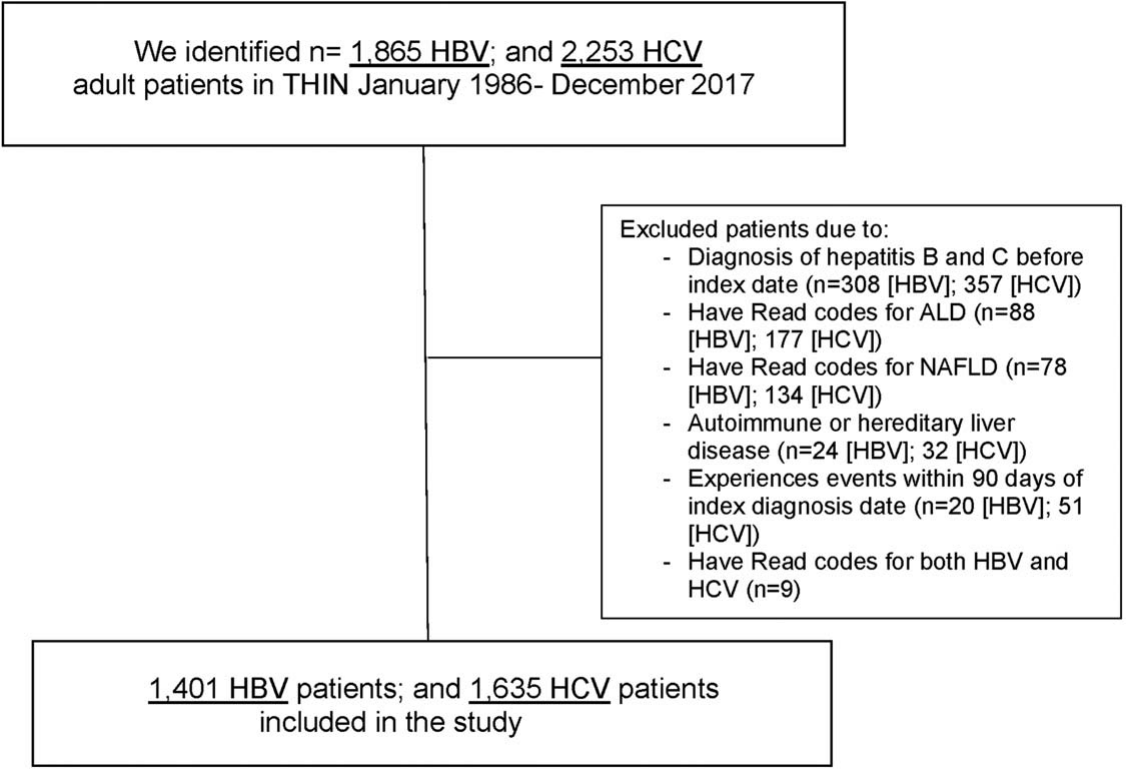
Flow diagram of patients in THIN included in the incident HBV or HCV cohorts. Abbreviations: ALD, alcohol-associated liver disease; THIN, The Health Improvement Network.

Using the THIN database, we included adults (≥18 y) with an incident diagnosis of HBV and HCV from January 1986 until December 2017. An incident case of HCV or HBV was defined as an individual who (1) had a diagnosis of HBV or HCV after registration in THIN and not within 90 days after their start date (physicians using THIN document history of previous diseases at registration before start date to list prevalent conditions) and (2) met the case definition for HBV or HCV (ie, >1 Read code for each condition recorded, separated by a time interval of ≥4 wk). Specific Read codes for each condition were used (Supplemental Table 1, http://links.lww.com/HC9/A155). The first code of HBV or HCV was considered the index date (ie, diagnosis date) for each case. We excluded patients who had diagnostic Read codes for other chronic liver disease (CLD). A flow diagram illustrating the selection of the study population is presented in Figure 1.

### Outcomes

Our composite primary outcome was the first occurrence of 1 of 2 events after an incidence diagnosis of HBV or HCV: (1) a clinical outcome indicating decompensation of cirrhosis as defined using validated Read codes; and (2) all-cause death.^14^,^18^–^21^ Each individual was followed from the index date of HBV or HCV diagnosis until either the development of clinical outcome of interest (ie, cirrhosis or death), migration from THIN database, or December 31, 2017, whichever came first. Read codes for HBV and HCV and specific decompensated cirrhosis outcome Read codes were externally validated in both the UK General Practice Research Database and THIN, and have shown high validity for identifying liver diseases and cirrhosis (Supplemental Table 1, http://links.lww.com/HC9/A155).^18^–^20^

### Exposure variables of interest

The primary exposures of interest were a diagnosis of MDD and the usage of antidepressants. Read codes were used to identify patients diagnosed with MDD (at least 1 code for MDD, as validated).^22^,^23^ MDD was dichotomized into (1) never diagnosed with MDD and (2) MDD diagnosis. A new diagnosis of MDD was captured only before the diagnosis of decompensated cirrhosis (ie, any diagnosis of MDD after the clinical outcome of interest was not analyzed).

THIN database therapy files were used to evaluate the impact of antidepressant medications on HBV or HCV outcomes. Similar to MDD, antidepressant use was classified into 2 groups: (1) medication was never used and (2) medication was used. Likewise, the prescriptions of antidepressants were restricted to before the diagnosis of decompensated cirrhosis. We assessed the effect of each of the following antidepressant medications on our study outcomes separately: atypical antidepressants, including agomelatine and bupropion and mirtazapine, were evaluated separately; mirtazapine has previously been associated with a protective effect in patients with primary biliary cholangitis.^14^ Antidepressants were characterized as: (1) selective serotonin reuptake inhibitors (SSRI); (2) selective-norepinephrine reuptake inhibitors; (3) serotonin modulators; and (4) tricyclics and tetracyclics.^24^

### Covariates

Demographic characteristics, including age at index diagnosis date of HCV or HBV (age was dichotomized as a categorical variable: <50 or ≥50 y at index date), sex, comorbid conditions, and body mass index were assessed. Comorbid conditions were evaluated using the Charlson Comorbidity Index to identify comorbid conditions within 3 years of enrollment in THIN and defined based on the presence of validated Read codes and associated weights.^25^

### Data analysis

Patient characteristics, classified by MDD status, were presented. Where appropriate, Chi-square and Kruskal-Wallis tests were used to identify statistical differences between the exposure groups at the study baseline. The incidence rates (IR) of primary composite outcome among HBV and HCV cohorts were calculated per 1000 person-years. All-cause mortality rates were calculated among each cohort per 1000 person-years. We used Poisson regression models to calculate the incidence rate ratio for MDD in each cohort.

Univariate analysis using the Log Rank test and time-dependent multivariate Cox Proportional Hazards models were implemented to assess the impact of MDD and antidepressants as time-varying covariates on composite primary outcome and mortality in both cohorts.^26^ This method accounts for the temporal relationship between exposure variables, covariates, and clinical outcomes (cirrhosis events or death) during the follow-up period, assessing the timing of MDD diagnosis or antidepressant prescription fill dates in relation to the timing of clinical outcomes. This method is recommended to avoid immortal time bias.^27^ The effect modification was explored for age and sex using a likelihood ratio test. Interaction terms were introduced and tested for depression and each class of antidepressant. Estimates were reported as HR and accompanying 95% CI.

Outcomes were evaluated in a propensity-matched analysis to control for selection bias based on an MDD diagnosis. Therefore, a propensity score for each MDD diagnosis in our cohorts was computed for clinically relevant predictors, including age, sex, and comorbidities. Propensity score matching was then conducted to match MDD diagnosis in each cohort in a 1:4 ratio for each cohort.^28^

### Sensitivity Analyses

Sensitivity analyses were done to evaluate the effect of the covariates on the relationship between primary exposures—MDD and antidepressants—and our composite primary outcome in the general cohorts without propensity score matching. We also examine the relationship between MDD and antidepressant use on mortality, regardless of decompensated cirrhosis status (mortality as a primary outcome). All analyses were performed using R Statistical Software version 3.3.1. Both the Conjoint Health Research Ethics Board at the University of Calgary and The Scientific Review Committee of THIN approved our study protocol (ID: 18THIN007).

## RESULTS

Between January 1986 and December 2017, we identified 1401 and 1635 incidents of HBV and HCV patients, respectively. MDD prevalence rates were higher among incident HCV (23.5%) compared with HBV (9.0%) patients, *p*<0.001. Similarly, antidepressant use was 2-fold higher among HCV patients than HBV patients (59.6% vs. 27.1%, *p*<0.001). Median follow-up for our cohorts was 5.6 years for HBV (interquartile range: 4.2–7.1) and 5.2 years for HCV (interquartile range: 4.8–5.3). Among the HBV cohort, patients with MDD were older (mean age 41 vs. 37, *p*=0.005) and had at least 1 comorbidity (30.1% vs. 17.6%, *p*<0.001; Table 1). Patients with HCV and MDD were less likely to be male (60.3% vs. 70.6%, *p*<0.001). There were no significant differences in age or comorbidities according to MDD status among the HCV cohort (Table 2). Antidepressant use was more common among MDD patients except for atypical antidepressants (similar distribution in both cohorts) and serotonin modulators (similar distribution among HCV cohort) (Tables 1 and 2).

**TABLE 1:** HBV cohort characteristics according to MDD diagnosis

Variables	No MDD (n=1275)	MDD (n=126)	*p*
Male sex, n (%)	715 (56.08)	66 (52.38)	0.48
Age, mean (SD)	37.41 (14.23)	41.08 (12.64)	0.005
Charlson Comorbidity Index,^a^ n (%)	—	—	0.001
0	1051 (82.43)	88 (69.84)	—
1	145 (11.37)	28 (22.22)	—
≥2	79 (6.2)	10 (7.94)	—
BMI, mean (SD)	25.49 (5.09)	26.2 (5.93)	0.17
Mirtazapine, n (%)	36 (2.82)	29 (23.02)	<0.001
Atypical antidepressants, n (%)	8 (0.63)	2 (1.59)	0.51
Serotonin modulators, n (%)	17 (1.33)	11 (8.73)	<0.001
SSRI, n (%)	204 (16.00)	90 (71.43)	<0.001
SNRI, n (%)	28 (2.20)	20 (15.87)	<0.001
TCA, n (%)	(13.88 177)	40 (31.75)	<0.001

^a^ Chronic liver disease was removed from Charlson Comorbidity Index for comparison.

Abbreviations: BMI, body mass index; MDD, major depressive disorder; SNRI, selective-norepinephrine reuptake inhibitors; SSRI, selective serotonin reuptake inhibitors; TCA, tricyclics and tetracyclics antidepressants.

**TABLE 2:** HCV cohort characteristics according to MDD diagnosis

Variable	No MDD (n=1250)	MDD (n=385)	*p*
Male sex, n (%)	882 (70.56)	232 (60.26)	<0.001
Age, mean (SD)	41.35 (12.21)	39.99 (10.60)	0.053
Charlson Comorbidity Index,^a^ n (%)	—	—	0.61
0	941 (75.28)	283 (73.51)	—
1	248 (19.84)	85 (22.08)	—
≥2	61 (4.88)	17 (4.42)	—
BMI, mean (SD)	24.86 (5.39)	24.86 (6.02)	1.00
Mirtazapine, n (%)	234 (18.72)	139 (36.10)	<0.001
Atypical antidepressants, n (%)	17 (1.36)	6 (1.56)	0.97
Serotonin modulators, n (%)	122 (9.76)	47 (12.21)	0.20
SSRI, n (%)	500 (40.0)	308 (80)	<0.001
SNRI, n (%)	74 (5.92)	60 (15.58)	<0.001
TCA, n (%)	302 (24.16)	136 (35.32)	<0.001

^a^ Chronic liver disease was removed from Charlson comorbidity index for comparison.

Abbreviations: BMI, body mass index; MDD, major depressive disorder; SNRI, selective-norepinephrine reuptake inhibitors; SSRI, selective serotonin reuptake inhibitors; TCA, tricyclics and tetracyclics antidepressants.

### Clinical outcomes

Among the HBV cohort, the IR of developing decompensated cirrhosis or death was 8.2 (95% CI, 6.4–10.6) per 1000 person-years. The IR was similar among HBV patients relative to their MDD status [no MDD: 8.3 (6.4–10.9) and MDD: 7.4 (3.3–16.4) per 1000 person-years, *p*=0.78; depression incidence rate ratio 0.89 (95% CI, 0.35–2.25)]. In the HCV cohort, the IR of decompensated cirrhosis or death was 22.6 (95% CI, 19.6–26.0) per 1000 person-years. The primary composite outcome IR was similar according to MDD status among HCV patients [no MDD: 22.4 (19.0–26.4); and MDD: 23.0 (17.3–30.5) per person-years, *p*=0.88; depression incidence rate ratio 1.05 (95% CI, 0.72–1.53)].

### Impact of MDD and antidepressants on decompensated cirrhosis-free survival

We evaluated the effect of MDD and antidepressants on both decompensated cirrhosis-free survival and mortality in propensity-matched cohorts of HBV and HCV according to age, sex, and comorbidities. In predicting decompensated cirrhosis-free survival among HBV and HCV patients, MDD was a nonsignificant predictor [adjusted HR (aHR): 0.60, 95% CI, 0.21–1.69; 1.13, 95% CI, 0.77–1.66, respectively] of our primary outcome. None of the antidepressants used was significantly associated with poor decompensated cirrhosis-free survival in patients with HBV infection (Table 3). However, among our HCV cohort, tricyclic/tetracyclic antidepressant (TCA) was a significant predictor of decompensated cirrhosis-free survival (aHR: 1.59, 95% CI, 1.13–2.23) (Table 3). Neither MDD nor any of the antidepressants was associated with morality in our propensity-matched HBV and HCV cohorts (Table 4).

**TABLE 3:** HRs (95% CI) for developing decompensated cirrhosis or mortality among HBV and HCV cohorts using propensity score-matched cohorts based on depression diagnosis

	HBV cohort propensity-matched cohort (n=111 with MDD diagnosis and n=444 without MDD diagnosis)		HCV cohort propensity-matched cohort (n=334 with MDD diagnosis and n=1018 without MDD diagnosis)	
Characteristic	Adjusted HR (95% CI)	Adjusted HR *p*	Adjusted HR (95% CI)	Adjusted HR *p*
MDD	0.60 (0.21–1.69)	0.33	1.13 (0.77–1.66)	0.52
Mirtazapine	2.11 (0.55–8.09)	0.28	1.04 (0.66–1.62)	0.88
Serotonin modulators	0.59 (0.06–5.84)	0.65	1.01 (0.58–1.74)	0.99
SSRI	1.66 (0.69–3.96)	0.26	1.03 (0.72–1.46)	0.88
SNRI	0.54 (0.06–4.65)	0.57	1.08 (0.59–1.98)	0.80
TCA	2.06 (0.95–4.46)	0.07	1.59 (1.13–2.23)	0.008
Sex				
Female	1.00 (reference)	—	1.00 (reference)	—
Male	1.39 (0.65–2.97)	0.40	1.89 (1.32–2.70)	<0.001
Age at start of follow-up (y)				
Under 50	1.00 (reference)	—	1.00 (reference)	—
Over 50	3.90 (1.66–9.15)	0.002	2.11 (1.49–2.98)	<0.001
Charlson Comorbidity Index^a^				
0	1.00 (reference)	—	1.00 (reference)	—
1	5.06 (1.78–14.36)	0.002	1.32 (0.92–1.90)	0.13
≥2	10.83 (3.47–33.80)	<0.001	1.59 (0.91–2.77)	0.10

^a^ Chronic liver disease was removed from Charlson comorbidity index for comparison.

Abbreviations: MDD, major depressive disorder; SNRI, selective-norepinephrine reuptake inhibitors; SSRI, selective serotonin reuptake inhibitors; TCA, tricyclics and tetracyclics antidepressants.

**TABLE 4:** HRs (95% CI) for mortality among HBV and HCV cohorts using propensity score-matched cohorts based on depression diagnosis

	HBV cohort propensity-matched cohort (n=111 with MDD diagnosis and n=444 without MDD diagnosis)		HCV cohort propensity-matched cohort (n=334 with MDD diagnosis and n=1018 without MDD diagnosis)	
Characteristic	Adjusted HR (95% CI)	Adjusted HR *p*	Adjusted HR (95% CI)	Adjusted HR *p*
MDD	0.52 (0.15–1.77)	0.30	1.09 (0.71–1.69)	0.70
Mirtazapine	3.31 (0.88–12.42)	0.08	0.93 (0.55–1.55)	0.77
Serotonin modulators	0.55 (0.05–5.76)	0.62	1.02 (0.54–1.92)	0.95
SSRI	1.43 (0.55–3.71)	0.46	1.58 (1.05–2.38)	0.028
SNRI	0.93 (0.10–8.36)	0.95	1.32 (0.70–2.50)	0.39
TCA	2.24 (0.99–5.06)	0.053	1.45 (0.98–2.15)	0.06
Sex				
Female	1.00 (reference)	—	1.00 (reference)	—
Male	1.14 (0.50–2.57)	0.76	1.72 (1.15–2.59)	0.009
Age at start of follow-up (y)				
Under 50	1.00 (reference)	—	1.00 (reference)	—
Over 50	4.19 (1.66–10.63)	0.003	3.49 (2.35–5.16)	<0.001
Charlson Comorbidity Index^a^				
0	1.00 (reference)	—	1.00 (reference)	—
1	7.70 (2.13–27.89)	0.002	1.19 (0.78–1.82)	0.42
≥2	16.68 (4.27–64.33)	<0.001	1.47 (0.78–2.74)	0.23

^a^ Chronic liver disease was removed from Charlson comorbidity index for comparison.

Abbreviations: MDD, major depressive disorder; SNRI, selective-norepinephrine reuptake inhibitors; SSRI, selective serotonin reuptake inhibitors; TCA, tricyclics and tetracyclics antidepressants.

### Sensitivity analysis

In our sensitivity analysis, evaluating all cohort patients, MDD was not associated with our primary outcome (aHR: 0.57, 95% CI, 0.23–1.39) in the HBV cohort. TCA were the only antidepressant group associated with poor decompensated cirrhosis-free survival (aHR: 1.80, 95% CI, 1.00–3.26) (Supplemental Table 2, http://links.lww.com/HC9/A155). Similarly, MDD was not a predictor of our composite primary outcome among HCV patients (aHR: 1.11, 95% CI, 0.78–1.59). Patients with HCV who used TCAs were more likely to have poor decompensated cirrhosis-free survival (aHR: 1.56, 95% CI, 1.13–2.14). None of the other antidepressants was associated with increased or reduced risk of the primary outcome (Supplemental Table 2, http://links.lww.com/HC9/A155). In both HBV and HCV cohorts, age and sex were not significant effect modifiers for the association between MDD or antidepressant usage and the study primary outcome. Furthermore, there were no significant interactions between antidepressants.

We evaluated the effect of MDD and antidepressants on mortality as a primary outcome. In our HBV cohort, similar to our main analysis, a diagnosis of MDD was not associated with increased mortality (aHR: 0.65, 0.24–1.75). Use of TCA was associated with a higher risk of mortality in the adjusted models (aHR: 2.18: 1.16–4.10) (Supplemental Table 3, http://links.lww.com/HC9/A155). Among the HCV cohort, sensitivity analysis for mortality as an outcome was similar to the HBV cohort, except for the use of an SSRI antidepressant (aHR: 1.48, 1.02–2.16), which was associated with higher mortality risk in the HCV cohort, whereas the use of TCA was not associated with an increased likelihood of death (aHR: 1.35, 0.93–1.96) (Supplemental Table 3, http://links.lww.com/HC9/A155).

## DISCUSSION

This population-based study describes the relationship between MDD, antidepressant use, and disease progression among patients with an incident diagnosis of HBV and HCV.

Decompensated cirrhosis and mortality rates were similar among patients with or without MDD in both of our HBV and HCV cohorts. Overall, in our adjusted analyses, having a diagnosis of MDD was not associated with developing decompensated cirrhosis or mortality among our incident HBV or HCV patient cohorts. However, the current use of TCAs was associated with poor decompensated cirrhosis-free survival in both cohorts. These findings were confirmed in our propensity score-matched cohort in HCV patients. Similar to our previous findings for patients with the autoimmune liver disease primary biliary cholangitis and alcohol-associated and NAFLD, a diagnosis of MDD did not impact disease progression or outcomes.^14^,^21^ Our current findings suggest increased mortality in HBV and HCV patients treated with tricyclic antidepressants. Although not statistically significant, we observed a similar trend among patients using TCA in the propensity-matched cohorts. These findings are important and may influence antidepressant choice in patients with chronic viral hepatitis.

The association between depression and CLD^29^,^30^ is well established, including for HBV and HCV.^7^,^8^ However, an association between the severity of liver disease in patients with HCV and HBV, and depression, is not well documented. In a study of 342,998 Korean adults followed for 7.8 years, 10,834 (3.16%) were HBsAg^+^, and 12.4% had depressive symptoms.^11^ Overall, those patients with depressive symptoms had an increased risk of liver-related mortality compared with those without depression. Moreover, the risk of liver-related mortality was significantly higher in HBsAg^+^ patients compared with HBsAg^−^ patients with depressive symptoms.

The longitudinal effect of antidepressant use on the progression of chronic viral hepatitis and the development of HCC was reported.^31^ Specifically, Chen et al^31^ examined the impact of TCAs and SSRIs, compared with no antidepressant treatment, on fibrosis progression and development of HCC among 128,201 HCV-infected veterans in the US followed for a mean of 7.2 years. Liver fibrosis progression was measured indirectly using the aspartate aminotransferase to platelet ratio score, and incident cases of HCC were recorded. Among the HCV-infected cohort, 4% were taking TCAs, 43% SSRIs, and 53% no antidepressants. Interestingly, persons taking TCAs had significantly less drug and alcohol misuse recorded than did SSRI users. The findings of this study cannot be generalized as (1) ≥95% of their cohorts were male, (2) alcohol misuse was ≥25%, and patients with alcohol-associated liver disease were not excluded, and (3) only 4% of their patients received TCA.^31^

In our cohort, we identified 1635 incident cases of HCV and 1401 incident cases of HBV. Similar to other reports, 23.5% of our HCV cohort had a diagnosis of MDD, as did 9.0% of our HBV cohort.^9^ Patients with MDD in our cohorts were more likely to be older (for both HBV and HCV), have more comorbidities (for the HCV cohort only), and to be female (for HCV but not HBV). For both our HCV and HBV cohorts, a diagnosis of depression did not significantly impact disease progression or overall mortality. In our adjusted models, only TCA use significantly increased the risk of mortality or developing decompensated cirrhosis in both of our HBV (aHR: 1.80; *p*=0.048) and HCV (aHR: 1.56; *p*=0.007) cohorts. For predicting mortality, TCA use significantly increased mortality risk in HBV (aHR: 2.18, *p*=0.016), whereas SSRIs increased that risk among HCV patients (aHR: 1.48, *p*=0.04). In our propensity-matched cohort to identify the independent predictors of the study outcomes, TCA antidepressant use was associated with developing decompensated cirrhosis or mortality among HCV patients (aHR: 1.59, *p*=0.008), and SSRI antidepressant use was associated with increased mortality risk in the same cohort (aHR: 1.58, *p*=0.028).

Prescribing antidepressants to CLD patients is often met with trepidation by many clinicians.^7^ Our findings have significant potential relevance to HCV and HBV patient care. Tri/tetracyclics have extensive first-pass hepatic metabolism, which can be significantly altered in advanced liver disease.^32^ This could potentially explain an increase in overall mortality with TCA use in chronic hepatitis patients; however, this explanation would not explain our finding of increased risk of decompensation with tricyclic/tetracyclic use in our HCV cohort, which suggests an adverse effect of these drugs on disease progression in HCV. This finding does not align with the report of Chen et al,^31^ which suggested a beneficial effect of tricyclics/tetracyclics on HCV-related disease progression. However, that study had multiple limitations, as described earlier. Moreover, the Chen study used an indirect measure of fibrosis progression (ie, AST to Platelets Ratio Index > 2.0), whereas we used the hard clinical endpoints of decompensation and mortality.^31^

Our current study suggests that TCAs should be used with caution in HCV-infected and HBV-infected patients and that SSRIs should be avoided in HCV-infected persons, especially if they have more advanced liver fibrosis and are at increased risk of decompensation. Our findings suggest that for patients with MDD and different liver disease etiologies, the choice of antidepressants should be guided by the potential differential impact of different antidepressant classes on disease outcomes and mortality.

Our study has several strengths, including a longitudinal retrospective cohort design allowing for the examination of the temporal associations between depression, antidepressant use, and clinical outcomes. Our patient cohorts were well defined using validated codes and included patients with multiple read codes to minimize misclassification risk. We also adjusted for important demographic and clinical variables that could potentially confound the relationship between depression, antidepressant use, and outcomes.

Our study also has some limitations. We used a case definition for MDD that was previously used and validated,^22^,^33^,^34^ but it does not allow us to ascertain the severity of depression. Specialist-prescribed medications such as biological therapy and direct-acting antiviral therapies were underreported in the THIN database.^20^ Therefore, we were not able to evaluate the impact of HBV or HCV treatments, such as interferon-based therapies. Similarly, we used a well-validated classification of antidepressants,^24^,^34^ but we cannot determine a dose-effect relationship for antidepressants, as the THIN database lacks validation tools and prescription duration and doses were missing for roughly 30% of patients. Therefore, we limited our definition of antidepressant use to patients who had confirmed antidepressant usage for ≥90 days. Future prospective studies with well-validated depression scales evaluating depression severity, and examining for antidepressant dose-effect relationships, are highly recommended.

In conclusion, we have shown in our well-defined longitudinal cohorts the effect of depression and antidepressant use on clinical outcomes, including patient survival, for HCV-infected and HBV-infected patients. Our findings suggest that the use of TCAs in HBV-infected and HCV-infected persons increases overall mortality and the risk of decompensation, and SSRI use in HCV-infected individuals increases their mortality risk. Given these findings, we recommend that the selection of a specific antidepressant should be personalized for patients with depression and CLD, based on the etiology of their liver disease, to optimize patient outcomes.

## Supplementary Material
