# Supplementary material for: Impact of depression and antidepressant use on clinical outcomes of hepatitis B and C: a population-based study
Source: Hepatol Commun. 2023 Feb 14;7(3):e0062. doi: 10.1097/HC9.0000000000000062 (PMC9931033; doi:10.1097/HC9.0000000000000062)
Supplement: Supplementary file 1 [file hc9-7-e0062-s001.docx]

Supplementary Table 1: Read codes of hepatitis B virus and hepatitis C virus cohorts and decompensated cirrhosis

| Read codes for hepatitis B virus | Read codes for hepatitis C virus | Read codes for decompensated cirrhosis |
| --- | --- | --- |
| A702.00 Viral hepatitis B with coma | 4JQD.00 Hepatitis C viral ribonucleic acid PCR positive | J622.11Encephalopathy - hepatic |
| A703.00 Viral hepatitis B without mention of hepatic coma | 4JQD.11 Hepatitis C PCR positive | G852000 Oesophageal varices with bleeding |
| A707000 Chronic viral hepatitis B with delta-agent | A704000 Viral hepatitis C with coma | G850.00 Oesophageal varices with bleeding else classified |
| A707100 Chronic viral hepatitis B without delta-agent | A705000 Viral hepatitis C without mention of hepatic coma | G85.11 Oesophageal varices |
| A707300 Chronic viral hepatitis B | A707200 Chronic viral hepatitis C | G851.00 Oesophageal varices without bleeding |
| A703000Acute hep B with delta-agent (coinfectn) without hep coma | A70z000 Hepatitis C | G852200 Oesophageal varices in cirrhosis of the liver |
| A705100Acute delta-(super)infection of hepatitis B carrier | ZV02C00 Hepatitis C carrier | 25O..00O/E Ascites |
| ZV02B00[V]Hepatitis B carrier | 2J11.00 Hepatitis C immune | 7H2B113 Insertion of peritoneal to venous shunt for ascites |
| 141E.00History of hepatitis B | 43X3.00 Hepatitis C antibody test positive | J624.00 Hepatorenal syndrome |
| 43B4.00Hepatitis B surface antig +ve | A705400 hepatitis non-A, non-B | SP14300 Hepatorenal syndrome as a complication of care |
| 43XA.00Hepatitis B core antibody positive |  | J554.00 Spontaneous bacterial peritonitis |
|  |  | B150300 Hepatocellular carcinoma |

Supplementary Table 2: Hazard ratios (95% CI) for developing decompensated cirrhosis or mortality among HBV or HCV cohorts

|  | HBV Cohort | | HCV Cohort | |
| --- | --- | --- | --- | --- |
| Characteristic | Adjusted HR  [95% CI] | Adjusted HR  p-value | Adjusted HR  [95% CI] | Adjusted HR  p-value |
| MDD | 0.57 (0.23-1.39) | 0.21 | 1.11 (0.78-1.59) | 0.57 |
| Mirtazapine | 1.55 (0.56-4.33) | 0.40 | 1.03 (0.68-1.57) | 0.89 |
| Serotonin modulators | 1.23 (0.41-3.74) | 0.71 | 1.13 (0.68-1.87) | 0.64 |
| SSRI^Ø^ | 1.76 (0.93-3.34) | 0.08 | 1.05 (0.76-1.46) | 0.76 |
| SNRI | 1.11 (0.36-3.42) | 0.86 | 1.05 (0.59-1.87) | 0.88 |
| TCA | 1.80 (1.00-3.26) | 0.048 | 1.56 (1.13-2.14) | 0.007 |
| Sex  Female  Male | 1.00 (Ref)  1.90 (1.06-3.40) | -  0.032 | 1.00 (Ref)  1.98 (1.41-2.79) | -  <0.001 |
| Age at start of follow-up  Under 50 years  Over 50 years | 1.00 (Ref)  4.20 (2.33-7.55) | -  <0.001 | 1.00 (Ref)  2.38 (1.74-3.27) | -  <0.001 |
| Charlson Comorbidity Index^φ^  0  1  ≥ 2 | 1.00 (Ref)  2.38 (1.23-4.58)  6.29 (3.23-12.25) | -  0.010  <0.001 | 1.00 (Ref)  1.50 (1.08-2.09)  1.58 (0.91-2.73) | -  0.016  0.11 |

^φ^ Chronic liver disease was removed from Charlson comorbidity index for comparison.

MDD, major depressive disorder; SSRI, selective serotonin reuptake inhibitors; SNRI, selective-norepinephrine reuptake inhibitors; TCA, tricyclics and tetracyclics antidepressants.

Supplementary Table 3: Hazard ratios (95% CI) for mortality among HBV and HCV cohorts

|  | HBV Cohort | | HCV Cohort | |
| --- | --- | --- | --- | --- |
| Characteristic | Adjusted HR  [95% CI] | Adjusted HR  p-value | Adjusted HR  [95% CI] | Adjusted HR  p-value |
| MDD | 0.65 (0.24-1.75) | 0.39 | 1.14 (0.76-1.71) | 0.53 |
| Mirtazapine | 2.18 (0.78-6.07) | 0.14 | 0.88 (0.54-1.45) | 0.62 |
| Serotonin modulators | 2.11 (0.73-6.07) | 0.17 | 1.04 (0.57-1.90) | 0.91 |
| SSRI | 1.35 (0.66-2.77) | 0.41 | 1.48 (1.02-2.16) | 0.04 |
| SNRI | 0.72 (0.16-3.24) | 0.67 | 1.33 (0.73-2.45) | 0.36 |
| TCA | 2.18 (1.16-4.10) | 0.016 | 1.35 (0.93-1.96) | 0.11 |
| Sex  Female  Male | 1.00 (Ref)  1.56 (0.83-2.90) | -  0.17 | 1.00 (Ref)  1.85 (1.26-2.73) | -  0.002 |
| Age at start of follow-up  Under 50 years  Over 50 years | 1.00 (Ref)  4.40 (2.25-8.60) | -  <0.001 | 1.00 (Ref)  3.83 (2.68-5.47) | -  <0.001 |
| Charlson Comorbidity Index^φ^  0  1  ≥ 2 | 1.00 (Ref)  3.56 (1.70-7.48)  9.41 (4.41-20.09) | -  <0.001  <0.001 | 1.00 (Ref)  1.34 (0.91-1.97)  1.41 (0.76-2.62) | -  0.14  0.27 |

^φ^ Chronic liver disease was removed from Charlson comorbidity index for comparison.

MDD, major depressive disorder; SSRI, selective serotonin reuptake inhibitors; SNRI, selective-norepinephrine reuptake inhibitors; TCA, tricyclics and tetracyclics antidepressants.
